# Screening and epitope characterization of diagnostic nanobody against total and activated *Bacteroides fragilis* toxin

**DOI:** 10.3389/fimmu.2023.1065274

**Published:** 2023-02-10

**Authors:** Yucheng Guo, Zhenlin Ouyang, Wenbo He, Jiaxin Zhang, Qian Qin, Min Jiao, Serge Muyldermans, Fang Zheng, Yurong Wen

**Affiliations:** ^1^ Center for Microbiome Research of Med-X Institute, The Key Laboratory of Environment and Genes Related to Disease of Ministry of Education, The First Affiliated Hospital, Xi’an Jiaotong University, Xi’an, China; ^2^ Laboratory of Cellular and Molecular Immunology, Vrije Universiteit Brussel, Brussels, Belgium

**Keywords:** *Bacteroides fragilis* toxin, metalloprotease, diagnosis, nanobody, epitope characterization

## Abstract

Enterotoxigenic *Bacteroides fragilis* (ETBF) can rapidly secrete an enterotoxin termed *B. fragilis* toxin (BFT), which is thought to be the only recognized virulence factor in ETBF. ETBF can cause acute diarrhea, inflammatory bowel disease (IBD), colorectal cancer, and breast cancer. BFT is divided into three subtypes, BFT1, BFT2, and BFT3. BFT1 is the most widely distributed in human *B. fragilis* isolates. BFT can be used as a biomarker for predicting the inflammation-cancer transformation of intestine and breast. Nanobodies have the advantages of small structure, complete antigen recognition capacity, rapid selection *via* phage display technology, and can be massively produced in microbial expression systems. Nanobodies have become a powerful tool for medical diagnosis and treatment. This study focuses on screening and structural characterization of nanobodies targeting full length and active BFT. By constructing prokaryotic expression systems to obtain recombinant BFT1 protein, high purity BFT1 protein was used to immunize alpacas. Phage display technology was used to construct a phage display library. The positive clones were selected by bio-panning, and the isothermal titration calorimetry was used to select high-affinity nanobodies. Then the three-dimensional structures of BFT1:Nb2.82 and BFT1:Nb3.27 were solved by crystal X-ray diffraction. We got two kinds of nanobodies, Nb2.82 targeting the BFT1 prodomain and Nb3.27 recognizing the BFT1 catalytic domain. This study provides a new strategy for the early diagnosis of ETBF and the possibility for BFT as a biomarker for diagnosing diseases.

## 1 Introduction

The gut microbiota occupies the large intestinal mucosa and is composed of tens of trillions of microorganisms. Microorganisms interact with the host in a bona fide manner and regulate multiple physiological processes, including pathogenicity, nutrient uptake, material metabolism, and host immunologic defense (1). *Bacteroides* genus is a ubiquitous member of the human gut microbiota. It can ferment sugars, starch, and fiber into volatile fatty acids, which are absorbed and used by the host cells. *B. fragilis* is considered the most virulent species and common clinical isolate in *Bacteroides* (2). *B. fragilis* is a conditional pathogen, divided into enterotoxigenic *B. fragilis* (ETBF) and non-enterotoxigenic *B. fragilis* (NTBF). ETBF can secrete an enterotoxin termed *B. fragilis* toxin (BFT), which is the most potent weapon of ETBF and encoded by the *bft* gene. The gene locates in the *B. fragilis* pathogenicity island (BfPAI), which is absent in NTBF strains (3).

ETBF can induce clinical pathology, including acute diarrhea, bacteremia, inflammatory bowel disease, and colorectal cancer (CRC). It is reported that ETBF stimulates rapid colitis and strongly induces colonic tumors in multiple intestinal neoplasia mice (4). Moreover, BFT induces ETBF tumorigenesis in *Apc^Min^* (Min: multiple intestinal neoplasia) mice (5). The predominant biofilm of familiar adenomatous polyposis (FAP) patients is mainly composed of polyketide synthase (*pks*)*^+^ E. coli* and ETBF. Co-colonization with *pks^+^ E. coli* and ETBF can increase colon tumor onset and mortality in Azoxymethane (AOM) mice (6). BFT is significantly occurring more often in mucosal samples from CRC patients than in healthy individuals. The positivity of BFT in late-stage CRC patients is higher than that in the early stage, where BFT is thought to be the high-risk factor for the late stage of CRC and can be a crucial biomarker for diagnosis (7). ETBF positivity and increased abundance are associated with early-stage carcinogenic lesions (8). Recent research also shows that ETBF can promote breast tumorigenesis and metastatic progression (9). Hence, it is crucial to detect and diagnose ETBF colonization. BFT is the only recognized virulence factor in ETBF (10), so it can be used as a biomarker for predicting inflammatory bowel disease, a risk factor for carcinogenesis, and the development of cancer.

BFT is a zinc-dependent metallopeptidase (11). Three different variants have been identified, including BFT1, BFT2, and BFT3 (12). BFT1 is widely represented in human *B. fragilis* isolates (13), BFT2 is confirmed to be the most toxic variant and can cause tissue damage, BFT3 has a geographical tendency in Southeast Asia (14, 15). The pre-protein of BFT consists of 397 amino acid residues with an 18-residue signal peptide for secretion, a 193-residue prodomain, and a 186-residue catalytic domain (16). The prodomain inhibits the catalytic domain activity *via* an aspartate-switch mechanism (16). The fragipain (Fpn) can cleave BFT-preproprotein into two discrete fragments, the auto-inhibitory prodomain is cleaved off, BFT is activated (10) and the catalytic domain is released from the bacterial cell.

The colonization of ETBF leads to occurrence of related diseases, so it is important to detect the active BFT. Colonization with ETBF is often determined by detecting the *bft* gene or its biological activity. The first method is to extract DNA directly from stool or surgical wound infection samples and the *bft* gene is detected *via* PCR and 16s rRNA (17). The stool and surgical wound infection samples contain PCR inhibitors, which reduce PCR sensitivity (18). In addition, when the *bft* gene does not express, or the samples contain dead bacteria, it is easy to generate false negative results. Moreover, the cost of PCR is expensive. The second method uses HT29/C1 cells (a human colonic epithelial tumor cell line) to detect the biological activity of BFT, which is secreted in the culture supernatant of ETBF strains (19). In a cytotoxic response of HT29/C1 cells, the cells exhibit morphological changes after BFT treatment, including cell rounding and dissolution of tight clusters (20). The main disadvantage of this method is the lack of objective criteria for morphological changes. Subjective factors of the experimenter easily influence the results. At the same time, this method involves the culture of HT29/C1 cells and the isolation and identification of anaerobic bacteria, which require complex experimental conditions and high technical skills. Other detection methods such as IMS-PCR (21), multiplex serology (22), and ELISA (23), monoclonal antibodies are required. Although monoclonal antibodies have high sensitivity and specificity, they suffer from several disadvantages such as their large molecular size, high production cost, short shelf-life (24), and eliciting immune reactions (25). Therefore, these methods of ETBF detection cannot be widely used in clinical diagnosis. It is necessary to develop a novel, convenient, accurate, sensitive and low-cost detection method.

Heavy chain antibodies (HCAbs) are naturally occurring and functional antibodies lacking light chains in camel serum. It contains a heavy chain variable region (VHH), and heavy chain constant regions 2 (CH2) and 3 (CH3). The VHH retains full antigen-binding capacity and is the smallest intact antigen-binding fragment. VHH is also named “nanobody” referring to their small size with nm dimensions (2.5 nm × 4 nm × 3 nm, 12~15 kDa) (26, 27). The discovery of nanobody opens a new era in antibody engineering, nanobodies have gradually become a potent tool in therapeutic drugs and clinical diagnostic reagents. Nanobodies have the advantages of a shorter preparation period, better stability, weak immunogenicity, specific antigen binding ability, and good tissue penetration (28). It has already been widely applied in molecular imaging, disease diagnosis, immunotherapy, and other fields. However, similar to most common anaerobic bacteria in human infection samples, *B. fragilis* related nanobodies have not been reported yet.

In this study, we focused on screening for specific BFT1 (full length without signal peptide) nanobodies. BFT1, the most widely distributed subtype, was used to immunize an alpaca. We have constructed a phage display library of specific nanobodies against the BFT1, and the library was used to retrieve the nanobodies. BFT1 nanobodies with high-affinity to antigen were assessed by isothermal titration calorimetry, then these nanobodies were co-crystallized with BFT1. We obtained nanobodies targeting the BFT1 prodomain (Nb2.82) and the BFT1 catalytic domain (Nb3.27), respectively. They form a powerful tool to detect mature and active BFT and provide a forecast for diagnosis of postoperative infection in patients. The study provides a new strategy for the early diagnosis of ETBF and enlightens the possibility of diagnosing CRC using BFT as a biomarker.

## 2 Materials and methods

### 2.1 Cloning, expression, and purification of recombinant BFT1

The DNA fragment was purchased (GENEWIZ, Suzhou, China) and cloned in the pET28a vector. The BFT1 plasmid was transformed into *Escherichia coli* Origami-2 (DE3) cells, protein expression was induced with 0.4 mM isopropyl β-D-1-thiogalactopyranoside (IPTG) at 18°C for 18–20 h, when the growth of culture reached an OD_600nm_ of 0.6. The BFT1 protein was purified by Ni-NTA chromatography followed by size exclusion chromatography (SEC) purification on a Superdex 75 column (GE Healthcare, Boston, USA). The purity of the protein was assessed by 12.5% SDS-PAGE stained with Coomassie blue. Recombinant BFT1 protein was used for alpaca immunization, bio-panning, and ELISA screening, mainly as described previously (29).

### 2.2 Library generation and bio-panning

An adult alpaca introduced from China was subcutaneously injected with 100 μg purified recombinant BFT1 protein plus adjuvant FAMA (Gerbu, Heidelberg, Germany) once a week. Peripheral blood was collected from jugular vein before the first injection and three days after the last injection, separated serum and compared immune effect by ELISA.

Three days after the last immunization, peripheral blood mononuclear cells (PBMCs) were extracted from alpaca blood, followed by the mRNA isolated from PBMCs. VHH was amplified by PCR and cloned in the vector pMES4 for phage display expression. The recombinant plasmid was transformed into TG1 cells by electroporation to construct a TG1 bacterial library, and the bacterial library was infected with M13K07 helper phage to obtain a VHH-phage displayed library. Phages expressing nanobodies with specificity for BFT1 were enriched after three rounds of bio-panning, following detailed experimental procedures as reported previously (29).

### 2.3 ELISA to quantitate initial binding

The bacterial library was established by infecting *E. coli* TG1 cells with the second and third rounds of phage libraries, individual bacterial colonies were randomly picked from the second and third rounds of bacterial libraries. These colonies were picked in 24-well plates and induced with 1 mM IPTG at 28°C for 18 h, when the growth of culture reached an OD_600 nm_ of 0.7. Wells of microtiter plates with 1 µgmL^-1^ BFT1 protein coating and blank wells were incubated with PBS overnight at 4°C. The periplasmic extracts were added after blocking residual protein binding sites in the wells with 2% skim milk in PBS and incubated for 2 h. After washing the wells, horseradish peroxidase (HRP)-conjugated His-tag antibody was added and incubated for 1 h. After washing, TMB substrate was added and to measure absorbance at 450 nm. The OD 450 nm signal of each well was divided by the signal of the well without antigen and considered positive if the resulting ratio was ≥ 2. These positive clones were chosen for DNA sequence determination.

### 2.4 Nanobody expression and purification

The plasmids containing the nanobody genes were transformed into *Escherichia coli* strain WK6 cells to express the nanobody in the periplasm of transformed cells. BFT1 nanobodies were extracted from periplasmic space by osmotic shock. All the extracted proteins were expressed in a 1000 mL medium. Nanobodies were purified by a Ni-NTA column and further purified by size exclusion chromatography (SEC) on a Superdex 75 column.

### 2.5 Isothermal titration calorimetry

A Microcal ITC 200 calorimeter (GE Healthcare, Boston, USA) was used to perform ITC experiments at 25°C. The BFT and nanobody proteins were exchanged to the same buffer (20 mM Tris pH 8.0, 150 mM NaCl, 5% glycerol). The nanobody protein (20 μM) was injected into a BFT solution (200 μM). The first injection was 0.4 μL and the second to 19^th^ injections were with 2 μL at 120 s intervals. The ITC data were analyzed with the supplemented Microcal ITC data analysis package under the one binding site mode.

### 2.6 Crystallization and data collection

The sitting-drop vapor diffusion method and 384-well plates were used for crystallization screening experiments and crystals appeared within 1 week at 20°C. The BFT1 and nanobody recombinant proteins were purified from size exclusion chromatography. Then, two kinds of proteins were concentrated and incubated (the molar ratio of BFT and nanobody is 1:1) for complex crystallization (30 mg mL^-1^). The BFT1:Nb2.82 complex crystals, which were observed in (1): 0.2 M potassium thiocyanate, 20% w/v PEG 3350 (2); 0.2 M lithium chloride, 20% w/v polyethylene glycol 3350, pH 6.8 (3); 0.2 M sodium malonate pH 5.0, 20% w/v polyethylene glycol 3350 (4); 8% v/v tacsimate pH 5.0, 20% w/v polyethylene glycol 3350. The BFT1:Nb3.27 complex crystals, which were observed in (1): 0.2 M ammonium tartrate dibasic, 20% w/v polyethylene glycol 3350, pH 6.6 (2). 2% v/v tacsimate pH 4.0, 0.1 M sodium acetate trihydrate pH 4.6, 16% w/v polyethylene glycol 3350. Crystals data were collected in the Shanghai Synchrotron Radiation Facility (SSRF) BL18U1 and BL19U1. The BFT1:Nb2.82 P212121 space group crystal diffracted to 1.66Å with α = β = γ = 90°, a = 55.663 Å, b = 78.212 Å, c = 118.132 Å. The BFT1:Nb3.27 C121 space group crystal diffracted to 2.25 Å with α = γ = 90°, β = 109.015°, a = 156.901 Å, b = 82.984 Å, c = 139.807 Å.

### 2.7 Structure determination

Diffraction images were processed using X-ray Detector Software (30). The structures of both BFT1:Nb2.82 and BFT1:Nb3.27 complexes were determined *via* molecular replacement using Phaser implemented in the Phenix package (31). The BFT3 structure (PDB: 3P24) was used as a search template for BFT1 (16). The structures of nanobodies were determined using Phaser implemented in the Phenix package using nanobody from 5IMM as a search model (32). The model was manually improved with the COOT program (33), and refinement was further done using Phenix refine (31). The interaction interface was calculated by PDB PISA (34). Moreover, figures were generated from PyMOL program, and data collection and refinement statistics are summarized in **Table 1**. Crystallographic coordinates and structure factors were deposited to the Protein Data Bank with access codes 8H3X and 8H3Y for BFT1:Nb2.82 and BFT1:Nb3.27, respectively.

**Table 1:** X-ray data collection and refinement statistics.

Crystal	BFT1:Nb2.82	BFT1:Nb3.27
Data collection		
Space group	P2_1_2_1_2_1_	C2_1_
a, b, c (Å)	55.66, 78.21, 118.13	156.90, 82.98, 139.80
α, β, γ (°)	90, 90, 90	90, 109.015, 90
Resolution (Å)	32.15-1.66 (1.72-1.66)	41.39-2.25 (2.33-2.25)
Rmerge	0.06499 (1.512)	0.1738 (1)
Rmeas	0.06774 (1.616)	0.2069 (1.201)
Multiplicity	12.3 (8.0)	3.4 (3.2)
CC (1/2)	1 (0.529)	0.984 (0.541)
CC*	1 (0.832)	0.996 (0.838)
I/σ(I)	21.83 (1.0)	8.40 (1.33)
Completeness (%)	99.48 (96.54)	98.52 (98.22)
Wilson B-factor (Å^2^)	29.19	41.31
Refinement		
Total Reflections	755356 (46719)	271519 (25498)
Unique Reflections	61429 (5862)	79575 (7917)
*R_work_*	0.1830	0.2039
*R_free_*	0.2163	0.2430
Number of atoms:		
Macromolecules	3796	11096
Ligands	1	3
Water	563	608
Average B-factor (Å^2^)	34.55	48.51
Protein (Å^2^)	33.57	48.64
Ligands	33.14	62.32
Water (Å^2^)	41.22	46.16
Ramachandran plot:		
Favored/Allowed (%)	98.5/1.3	97.6/2.2
Root-Mean-Square-Deviation:		
Bond lengths (Å)	0.007	0.009
Bond Angle (°)	1.04	1.03

Statistics for the highest resolution shell are shown in parentheses. CC* = [2 CC(1/2)/(1 + CC(1/2))]^1/2^.

## 3 Results

### 3.1 Immune effectivity evaluation of recombinant BFT1

**Figure 1:**
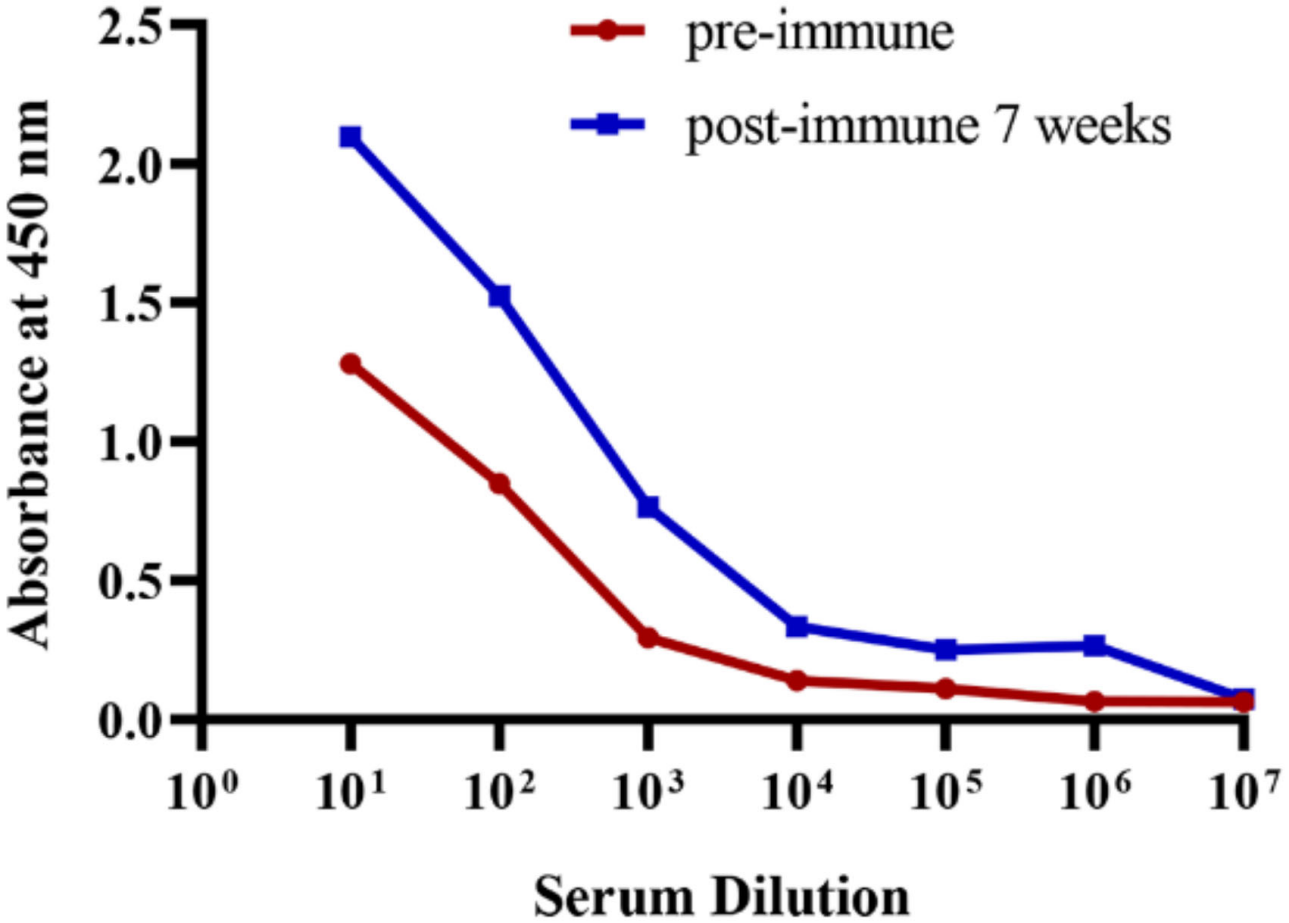
ELISA analysis of the BFT1 specific antibody titers from pre-immune and post-immune (7 weeks) alpaca serum.

Recombinant BFT1 was produced in an *E. coli* prokaryotic expression system and used as antigen. Before immunization, the integrity and purity of BFT1 protein were tested by SDS-PAGE and Coomassie blue staining (**Supplementary Figure 1**). An alpaca was immunized seven times at a weekly interval. Enzyme-linked immunosorbent assay (ELISA) was used to detect the BFT1-specific antibody titers from pre-immune and post-immune alpaca serum (**Figure 1**). Compared with pre-immunization, BFT1-specific antibody titers increased after seven times of immunization.

### 3.2 BFT1 nanobody generation

Three days after the last immunization, blood was collected from the jugular vein, and total RNA was extracted from the peripheral blood lymphocyte cells (PBLs). RT-PCR generated the cDNA, and nested PCR amplified the VHH gene fragment. The immune VHH library was constructed after ligating of the VHH fragments in the pMES4 vector. After the transformation of recombinant phagemids into *E. coli* TG1 cells, we successfully obtained a library with a size of 1.93×10^7^ individual clones. Recombinant BFT1 protein was coated in microplate wells as antigen, and phage display panning was the method to retrieve BFT1-specific nanobodies. After three rounds of phage display panning, BFT1-specific nanobodies were enriched (**Table 2**). After three rounds of bio-panning for BFT1, a gradual increase in enriched phages from 1.6×10^9^ to 2×10^12^ cfu mL^-1^ was noted.

**Table 2:** Enrichment of phages with each consecutive round of bio-panning.

Round	Input (cfu mL^-1^)	BFT1 output(cfu mL^-1^)	PBS output(cfu mL^-1^)	Recovery (BFT1/input)	BFT1/PBS
1	4.8×10^11^	1.6×10^9^	4×10^6^	3.3×10^-3^	400
2	2.5×10^14^	1×10^12^	3.6×10^6^	4×10^-3^	2.7×10^5^
3	1.2×10^14^	2×10^12^	1×10^8^	1.67×10^-2^	2×10^4^

Input corresponds to the number of phages incubated in wells and output corresponds to phages eluted after each round of bio-panning. cfu (colony forming units).

In order to ensure the diversity of nanobodies, 188 individual clones were randomly picked after the 2nd round (94 colonies) and 3rd round (94 colonies) of bio-panning. The individual clones were grown and nanobodies were induced and expressed with IPTG, and periplasmic extracts were tested in ELISA against recombinant BFT1 protein. 135 individual clones were considered positive colonies and sent for sequencing since the ratio between the signal in wells with coated antigen and background signal was more than 2-fold (**Table 3**).

**Table 3:** PE-ELISA of individual colonies screened for binding to BFT1 from panning of round 2 and 3.

Specific signal/background signal	Round 2	Round 3	Total
> 2-fold	83	52	135
< 2-fold	11	42	53

188 individual phage clones selected from panning of round 2 and 3. The number of colonies were calculated by PE-ELISA, and the values were measured at 450 nm. >2-fold colonies were DNA sequenced.

**Figure 2:**
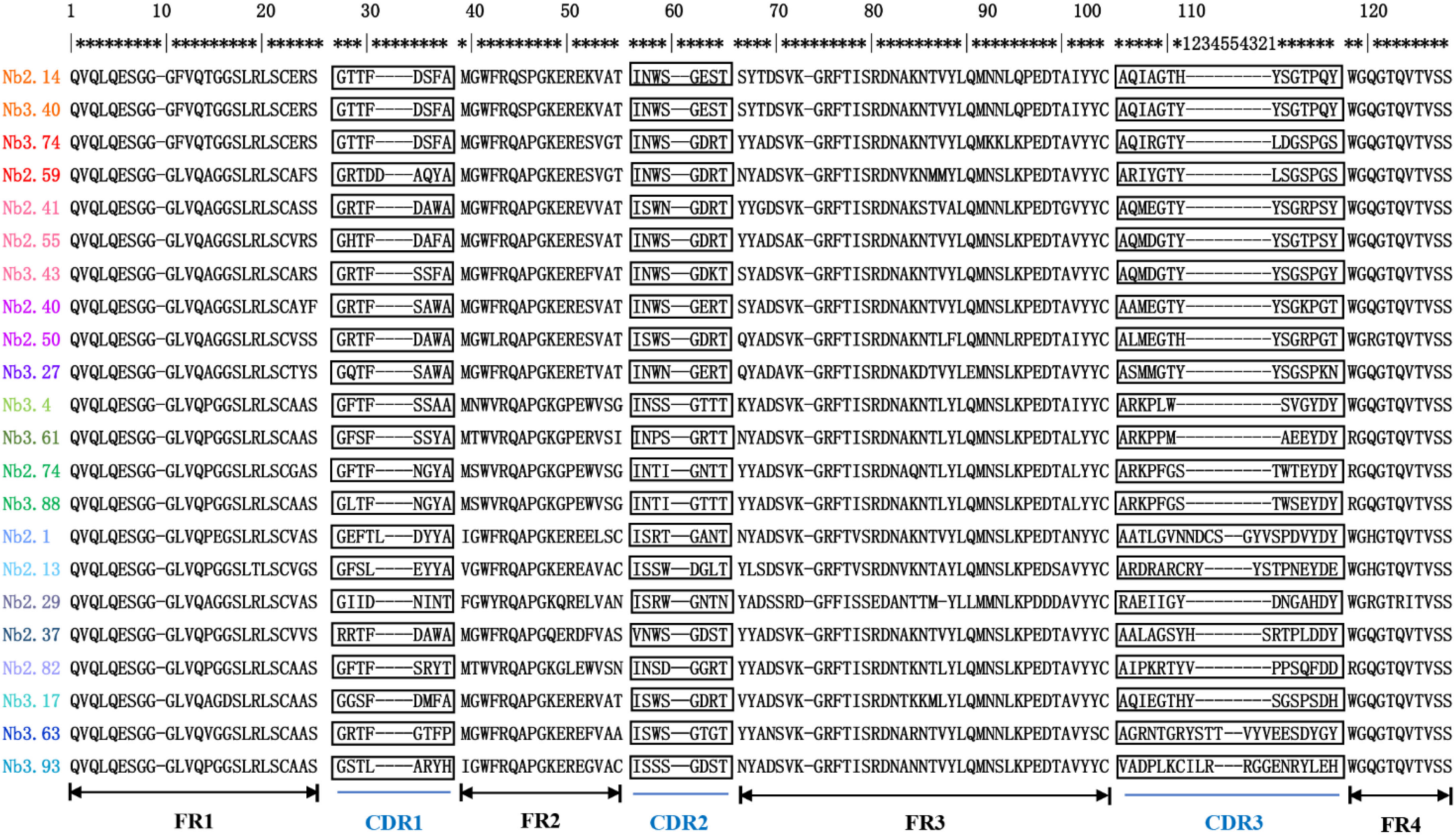
The amino acid sequence of BFT1 nanobodies. Framework regions (FR) and complementarities determining regions (CDR) were determined by blasting against the IMGT database. CDR sequences are marked with black rectangles, the same group is marked with the same color.

The VHH contains four framework regions (FRs) and three antigen-binding complementarity determining regions (CDRs). The CDR3 loop of VHHs is the major contributor for antigen interaction and prefers to recognize holes and cavities in the spatial conformation of antigens. Sequence diversity in the CDR3 of VHH is sufficient for most antibody specificities. According to the international immunogenetics information system (IMGT), the framework and complementarity determining regions of the nanobodies were identified. Among 135 positive clones, 22 distinct VHH fingerprints were identified, belonging to 16 different groups based on their unique CDR3 (**Figure 2**). The amino acid sequences of CDR1, CDR2, and FRs were also different among different groups. These 22 nanobodies were positive representative clones (**Figure 2**). All the nanobodies were expressed with a C-terminal His_6_-tag for purification.

### 3.3 Expression and purification of BFT1 nanobodies

**Figure 3:**
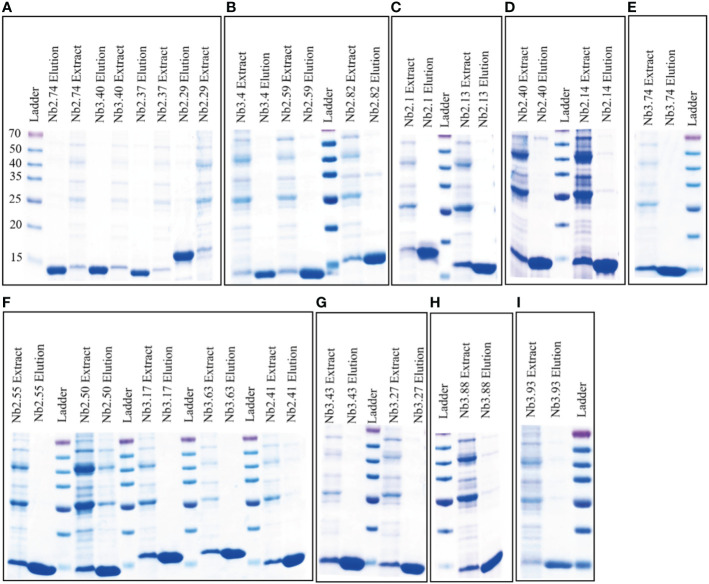
Coomassie-stained 12.5% SDS-PAGE gels were used to analysis the purity and size of BFT1 nanobodies. The results revealed BFT1 nanobodies with molecular size 14 kDa. **(A)** Nb2.74, Nb 3.40, Nb2.37, Nb2.29 **(B)** Nb3.4, Nb2.59, Nb2.82 **(C)** Nb2.1, Nb2.13 **(D)** Nb2.40, Nb2.14 **(E)** Nb3.74 **(F)** Nb2.55, Nb2.50, Nb3.17, Nb3.63, Nb2.41 **(G)** Nb3.43, Nb3.27 **(H)** Nb3.88 **(I)** 3.93. Ladder: relative molecular mass marker (kDa); Extract: extract from periplasmic; Elution: 500 mM imidazole-Tris.

Nanobody containing plasmids were transformed into *E. coli* strain WK6 cells and nanobody protein was expressed in the periplasm of cells. Soluble nanobodies with His_6_-tag were extracted from the periplasm and affinity purified using nickel beads. The purity and size of BFT1 nanobodies were analyzed by Coomassie-stained 12.5% SDS-PAGE gels (**Figure 3**). The lane of elution showed high purity and the molecular weight of nanobodies around 14 kDa. These were consistent with the size calculated from amino acid sequences. The yield of BFT1 nanobodies varied from 2.5 mg to 34 mg per liter (**Supplementary Table 1**).

### 3.4 The interactions of nanobodies with BFT1 by isothermal titration calorimetry

**Figure 4:**
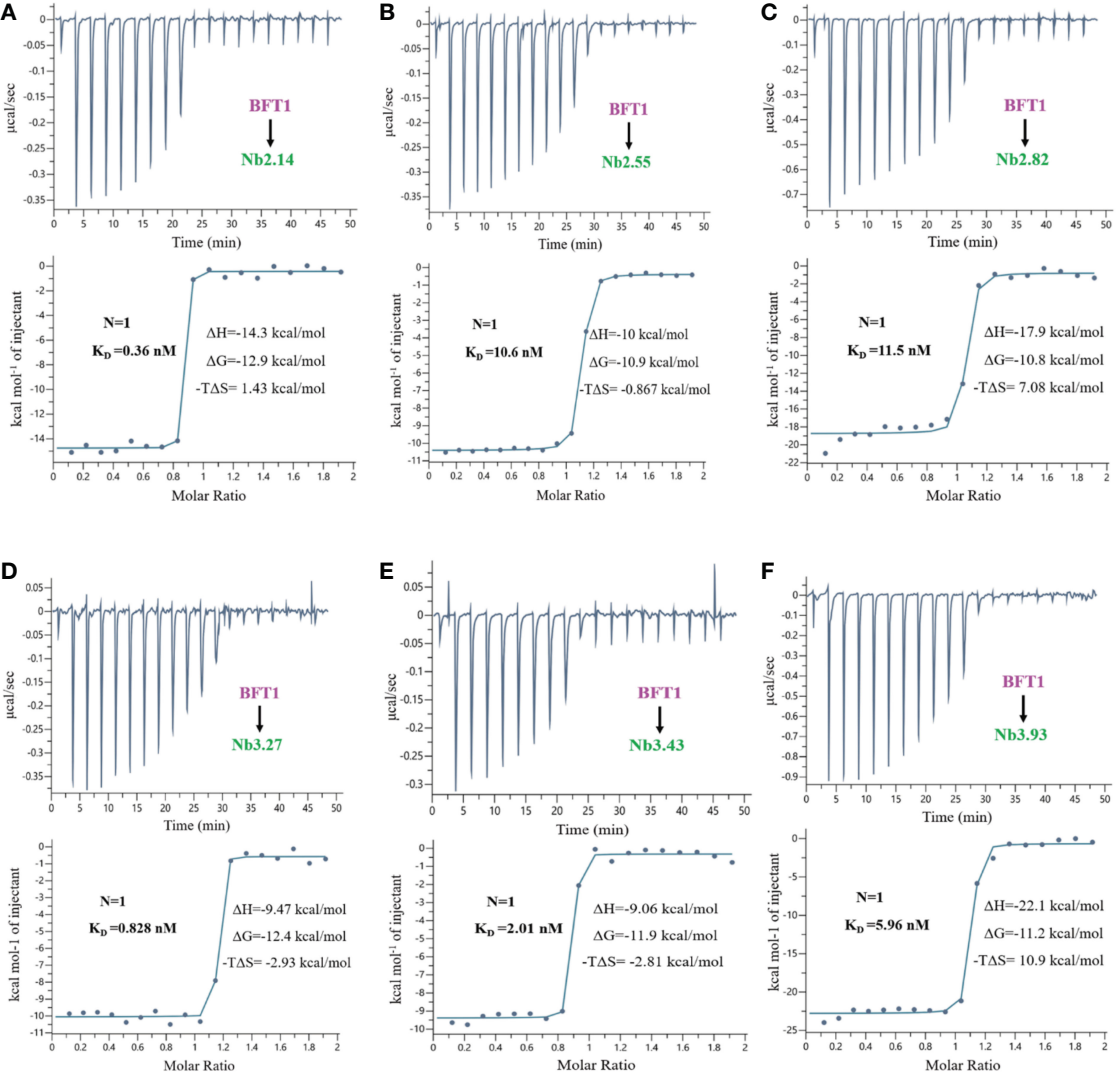
The isothermal titration calorimetery of BFT1 and nanobody. There were 6 nanobodies with high affinity of BFT1, **(A)** Nb2.14 **(B)** Nb2.55 **(C)** Nb2.82 **(D)** Nb3.27 **(E)** Nb3.43 **(F)** Nb3.93. Dissociation constant (K_D_), stoichiometry (N), enthalpy (ΔH), entropy (ΔS) and Gibbs free energy (ΔG) are denoted.

The proteins of nanobodies and recombinant BFT1 were purified by nickel column affinity chromatography, and size exclusion chromatography profile indicates that BFT1 can form complexes with nanobodies (**Supplementary Figure 2**). The stoichiometry and thermodynamics of the interactions of nanobodies with BFT1 were characterized by isothermal titration calorimetry (**Table 4**). **Figure 4** and **Supplementary Figure 3** show that an exothermic reaction occurred when the nanobody interacted with BFT1. The molar ratio of nanobody to BFT1 protein is 1: 1. The affinity of antibodies is described by the dissociation constant (K_D_). The smaller the K_D_ value, the higher the affinity between the antibody and its antigen. Fourteen different nanobodies against BFT1 had an affinity ranging from 0.36 to 11.5 nM, the affinities of Nb2.14, Nb3.17, and Nb3.27 were between 0.36~0.828 nM, the affinities of 9 nanobodies (Nb2.29, Nb2.41, Nb3.43, Nb3.40, Nb3.63, Nb2.37, Nb3.4, Nb3.93, and Nb2.40) were between 1.18~8.72 nM, whereas the affinities of Nb2.55 and Nb2.82 were 10.6 nM and 11.5 nM.

**Table 4:** The stoichiometry and thermodynamics binding constants of BFT1 nanobodies.

NO	Name	Round	K_D_ (nM)	ΔH (kcal/mol)	ΔG (kcal/mol)	-TΔS (kcal/mol)
1	Nb2.14	2	0.36	-14.3	-12.9	1.43
2	Nb2.29	2	1.18	-15.5	-12.2	3.35
3	Nb2.37	2	3.91	-14.1	-11.5	2.6
4	Nb2.40	2	8.72	-13.5	-11	2.51
5	Nb2.41	2	1.37	-20	-12.1	7.89
6	Nb2.55	2	10.6	-10	-10.9	-0.867
7	Nb2.82	2	11.5	-17.9	-10.8	7.08
8	Nb3.4	3	3.94	-8.4	-11.5	-3.07
9	Nb3.17	3	0.66	-20.3	-12.5	7.75
10	Nb3.27	3	0.828	-9.47	-12.4	-2.93
11	Nb3.40	3	2.58	-13.6	-11.7	1.92
12	Nb3.43	3	2.01	-9.06	-11.9	-2.81
13	Nb3.63	3	3.37	-18.8	-11.6	7.24
14	Nb3.93	3	5.96	-22.1	-11.2	10.9

### 3.5 Overall structures of nanobodies in complex with BFT1

**Figure 5:**
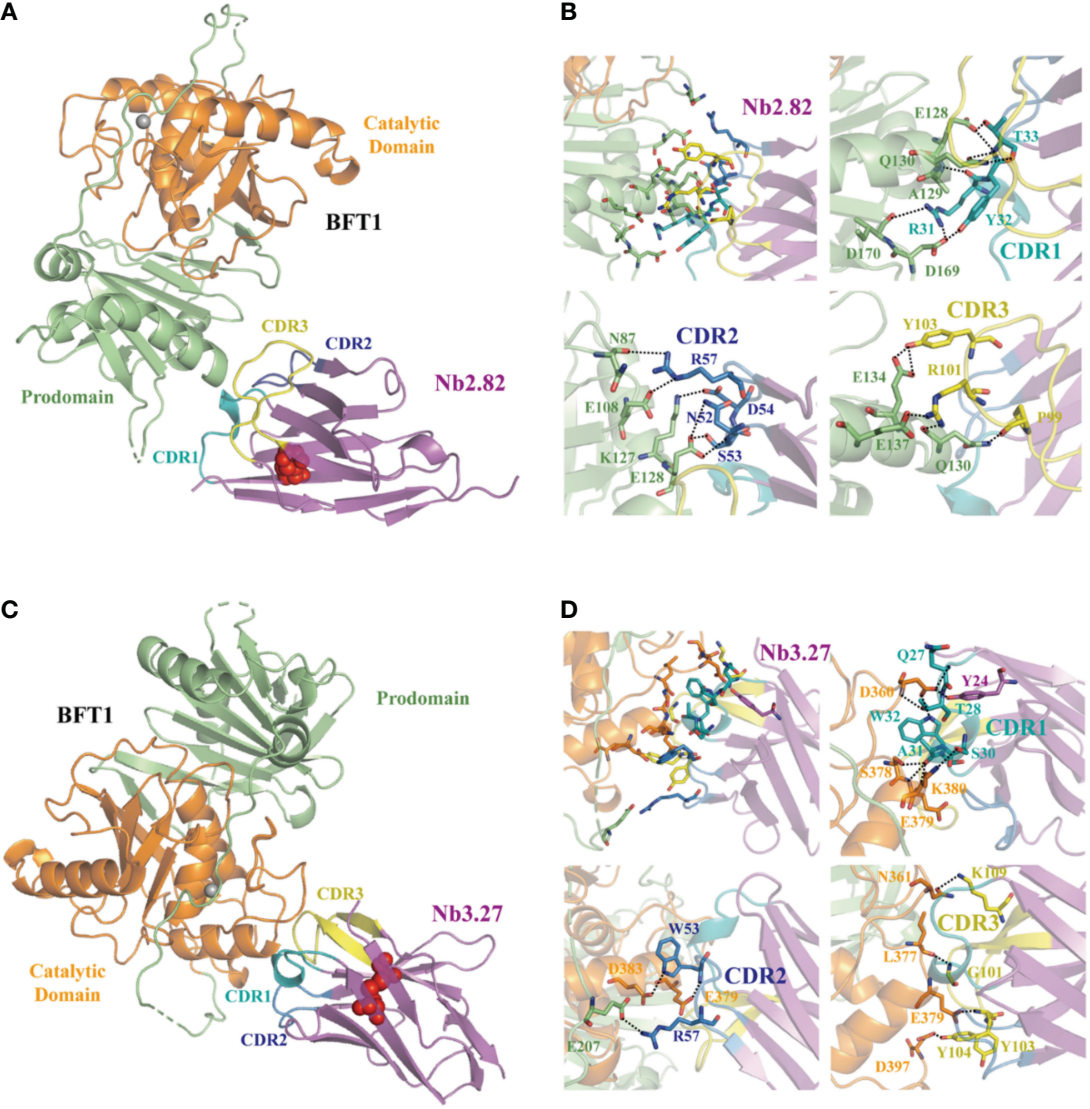
The structures of nanobodies in complex with BFT1. The overall crystal structures of nanobodies Nb2.82 and Nb3.27 in complex with BFT1 are shown in **(A, C)** respectively. The nanobodies are colored magenta, BFT1 prodomain is colored in lime, BFT1 catalytic domain is colored in orange. The CDR1, CDR2, CDR3 are shown in the color teal, skyblue and yellow, the cysteine involved in disulfide bonding forming are denoted in red sphere. The magnified views of the interaction region are shown in **(B, D)** respectively. The color mode is the same as in **(A, C)**, residues involving in interaction are shown as stick.

Fourteen high-affinity nanobodies were separately crystallized with BFT1. Fortunately, we obtained two complex crystal structures, BFT1:Nb2.82 and BFT1:Nb3.27. Nb2.82 targets BFT1 prodomain and Nb3.27 targets BFT1 catalytic domain. The crystallographic structure of the BFT1:Nb2.82 crystals in space group P212121 was solved to 1.66 Å and that of BFT1:Nb3.27 in space group C121 was solved to 2.25 Å. Data collection and refinement statistics are reported in **Table 1**. The BFT1 and nanobody can form a stable and compact heterodimer. The CDR regions of nanobodies are directly involved in antigen binding, these regions consist of two antiparallel β-sheets, and the overall structures of Nb2.82 and Nb3.27 contains ten β-strands to form two β-sheets which are connected by a disulfide bond between Cys22 and Cys96. The CDR1 to 3 of Nb2.82 has 8 residues (Gly26 to Thr33), 8 residues (Ile51 to Thr58) and 15 residues (Ala97 to Asp111), respectively (**Figure 5A**). The Nb3.27 has CDR1 to 3, comprising 8 residues (Gly26 to Ala33), 8 residues (Ile51 to Thr58), and 14 residues (Ala97 to Asn110), respectively (**Figure 5C**). The CDR1, 2, and 3 loops of Nb2.82 mainly recognize the α-helix in BFT1 prodomain (**Figure 5B**), while the CDR1, 2, 3 loops of Nb3.27 mainly recognize the α-helix in BFT1 catalytic domain (**Figure 5D**). The amino acids of the CDR1 loop of Nb2.82 form 8 hydrogen bonds and one salt bridge with BFT1, the CDR2 forms 6 hydrogen bonds and 3 salt bridges, and the CDR3 forms five hydrogen bonds, and two salt bridges. The amino acids of the CDR1 loop of Nb3.27 form 8 hydrogen bonds with BFT1, the CDR2 forms three hydrogen bonds and one salt bridge and the CDR3 forms four hydrogen bonds. The detailed interaction interface between BFT1 and nanobodies are listed in **Supplementary Table 2** and **Table 3**.

## 4 Discussion

An increasing number of studies have shown that the gut microbiota is essential for maintaining host health. The disturbance of the gut microbiota is closely related to various diseases. ETBF is an intestinal anaerobic bacterium, it can live in the intestine without causing symptoms, but it also can cause diarrhea in livestock and humans. Comparing laterally spreading tumor patients with healthy controls, the abundance of ETBF in fecal samples is increased 9.26-fold (35). ETBF secretes *B. fragilis* toxin (BFT) that targets the colonic epithelium to trigger an IL-17 mucosal immune response, then IL17 activates NF-κb and stat3 signaling pathways, induces the release of signaling molecules (CXC chemokines) and recruits more immune cells to participate in the inflammatory response to promote tumorigenesis (5). ETBF is also colonized in the mammary gland, promotes breast tumorigenesis, and activates Notch and β-catenin axes (9). In addition, *B. fragilis* is the most ordinary pathogen in patients’ infections after the gastrointestinal operation. *B. fragilis* bacteremia causes high mortality, particularly among older people and cancer patients (36).

The current detection methods of BFT with conventional antibodies have problems of being expensive, subjective interpretation, poor stability, and low yield. Moreover, these approaches cannot detect the stage of BFT. Therefore, it is necessary to design a novel, rapid, accurate, sensitive, robust, and low-cost diagnostic method for detecting the total and active BFT. Nanobodies are generally regarded as a substitute for conventional antibodies because of their unique structure and chemical stability and have become a new research tool for disease diagnosis and treatment. Nanobodies are also referred to as VHH, and there are three main differences between VHH and the VH domain of a conventional antibody. First, a VHH usually has an enlarged CDR1, and CDR3 (37). The loss of the light chain leads to a decrease in antibody binding capacity. However, the expansion of CDR1 and CDR3 can largely compensate for this theoretical loss. Because the extended CDR3 can form a finger-like structure or a convex paratope that penetrates the interior of the antigen to bind to antigenic epitopes that are much less antigenic for conventional antibodies (24). Therefore, the affinity of a nanobody for its cognate antigen can be as high or even higher than that of conventional antibodies. Second, a notable difference between VHH and VH is that the four highly conserved hydrophobic residues (V42, G49, L50, W52, IMGT numbering) in the VH framework-2 region are replaced by hydrophilic residues (often F42, E49, R50, G52) in VHH (38), thereby VHH can maintain good specificity and affinity, and the solubility and stability have been increased. Third, an additional interloop disulfide bond between CDR1 (or FR2) and CDR3 in VHH is often observed in VHH, reducing the entropic penalty for nanobody-antigen binding, increasing the conformational stability of nanobody, enhancing heat and acid-alkali resistance of nanobodies (39). Because of the above-mentioned structural characteristics, nanobodies have the advantages of both conventional antibodies and small molecule drugs. The nanobodies can be used for developing clinical diagnostic kits because of the following characteristics: lower production cost, easy modification to improve specificity and affinity, high stability against elevated temperature, organic solvents or non-physiological conditions, targeting hidden epitopes, and low immunogenicity (28). Nanobodies have gradually become an emerging force in therapeutic drugs and clinical diagnostic reagents.

In this study, we aimed to screen the nanobodies targeting BFT1, providing a new idea and scientific support for developing diagnostic methods of ETBF. According to the research of Theodoros Goulas (16), we expressed and purified the recombinant BFT1 protein in a prokaryotic expression system. The high purity and correct size recombinant protein was used to immunize alpacas. After seven immunizations, a high titer of BFT1-specific antibodies was obtained, confirming that the recombinant BFT1 was a good immunogen in experimental animals. We obtained the VHH library with a size of 1.93×10^7^ individual clones, and the library capacity is similar to our previous study (40). Individual phage clones have different binding abilities to an antigen, hence the high capacity and diversity of the library are the prerequisites for screening the specific and high-affinity nanobodies (41). After three rounds of bio-panning, BFT1-specific phages were significantly enriched, and the number of phages increased from 1.6×10^9^ to 2×10^12^ c.f.u mL^-1^. To ensure the diversity of sequences, we selected 135 individual clones for DNA sequencing and finally obtained 22 amino acid sequences with unique CDR3. This result also reflects the diversity of the library. The clones were cultured in 1 L medium, and the yield of BFT1 nanobodies varied from 2.5 mg to 34 mg per liter. It is normal for nanobodies to have large differences in expression (40, 42). Isothermal titration calorimetry was used to quantify the interactions between nanobodies with BFT1. The dissociation constant (K_D_) varied from 0.36 nM to 11.5 nM. This affinity of nanobodies for their antigen is comparable with other publications (38).

We crystallized high-affinity nanobodies with recombinant BFT1 separately, the crystal structures were studied by X-ray diffraction, and the structures of the complexes BFT1:Nb2.82 and BFT1:Nb3.27 were obtained. The CDR loops of Nb2.82 mainly recognize the α-helix in the BFT1 prodomain, while Nb3.27 mainly interacts with the α-helix in BFT1 catalytic domain. Disulfide bonds in nanobodies are classified into conserved, and additionally almost all structures of nanobodies contain a conserved disulfide bonds linked by FR1 (Cys22) and FR3 (Cys96). A conserved disulfide bond connects the two β-sheets to increase structural stability. However, only some nanobodies contain additional disulfide bonds, and their location and number are also different in different nanobodies. These mainly depend on the position and number of cysteines in nanobodies. The additional disulfide bonds can limit CDR loop flexibility and conformation. Nb2.82 and Nb3.27 only contain two cysteines (Cys23 and Cys104), and they just have conserved disulfide bonds. Moreover, the CDR3 loops of the nanobodies fold over some of the FR2 residues to make them solvent inaccessible, which will prevent nanobody stickiness and dimerization.

Nb2.82 and Nb3.27 can detect the total and activated BFT in human tissue or feces by enzyme-linked immunosorbent assay. Nb2.82 and Nb3.27 contain His_6_-tag, which can be used as detection antibodies. The tagged protein binds to the corresponding enzyme-labeled antibody. After the substrate is added, the antigen and small molecules are detected by enzyme-catalyzed substrate coloration. We can also use nanobodies and biotin-streptavidin ELISA to improve the detection sensitivity. Yaozhong Hu and colleagues (43) developed a sandwich ELISA by employing the nanobody and biotinylated-nanobody pair to capture and detect *Staphylococcus aureus* in milk. Moreover, Linzhi Li (44) developed a novel ultrasensitive electrochemiluminescence (ECL) immunoassay based on nanobody and Au/CaCO_3_, which was proposed for detecting ochratoxin A in coffee. Nb2.82 and Nb3.27 can also be integrated with electrochemical detection technology to determine BFT rapidly.

In conclusion, we have revealed two nanobodies that recognize distinct epitopes on BFT1, Nb2.82 targets BFT1 prodomain and Nb3.27 targets BFT1 catalytic domain. We are the first to obtain *B. fragilis*-related nanobodies. Future work on these nanobodies will develop detection kits to quantify the active BFT in patients and healthy people, providing judgment methods for the prevention of intestinal diseases and postoperative intervention of patients.

## Data availability statement

Crystallographic coordinates and structure factors were deposited to the Protein Data Bank with access codes 8H3X and 8H3Y for BFT1:Nb2.82 and BFT1:Nb3.27, respectively. The datasets presented in this study can be found in online repositories. The names of the repository/repositories and accession number(s) can be found in the article/**Supplementary Material**.

## Ethics statement

All applicable institutional and/or national guidelines for the care and use of animals were followed and all animal experiments were approved by the Ethical Committee for Animal Experiments of Xi’an Jiaotong University (NO. XJTU-2020-35).

## Author contributions

YW, FZ, YG designed the experiments. YG, ZO, JZ, and QQ performed the characterization experiments. YG, ZO, WH, MJ and YW performed crystallography experiments. YG, WH, YW and FZ, SM analysed data. YG and YW wrote the manuscript. All authors contributed to the article and approved the submitted version.
