# Supplementary material for: Screening and epitope characterization of diagnostic nanobody against total and activated Bacteroides fragilis toxin
Source: Front Immunol. 2023 Feb 10;14:1065274. doi: 10.3389/fimmu.2023.1065274 (PMC9950733; doi:10.3389/fimmu.2023.1065274)
Supplement: Supplementary file 1 [file DataSheet_1.pdf]

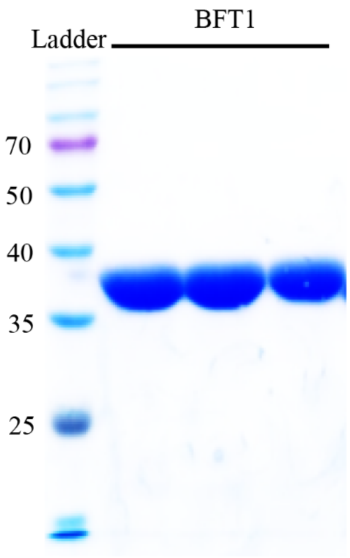

Supplementary Figure 1. Production of recombinant BFT1. SDS-Page to ensure protein integrity and purity of BFT1 (without signal peptide) before immunization.

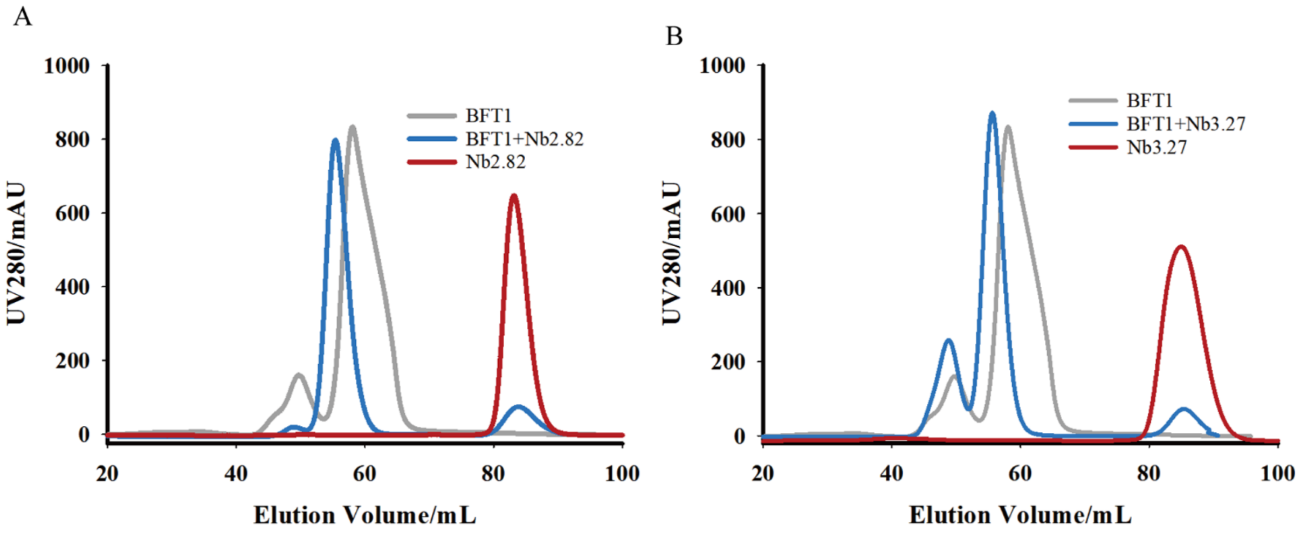

Supplementary Figure 2. BFT1 formed complexes with **(A)** Nb2.82 or **(B)** Nb3.27 by size exclusion chromatography. BFT1:Nb2.82 and BFT1:Nb3.27 complexes were purified by a superdex 75 column (GE Healthcare).

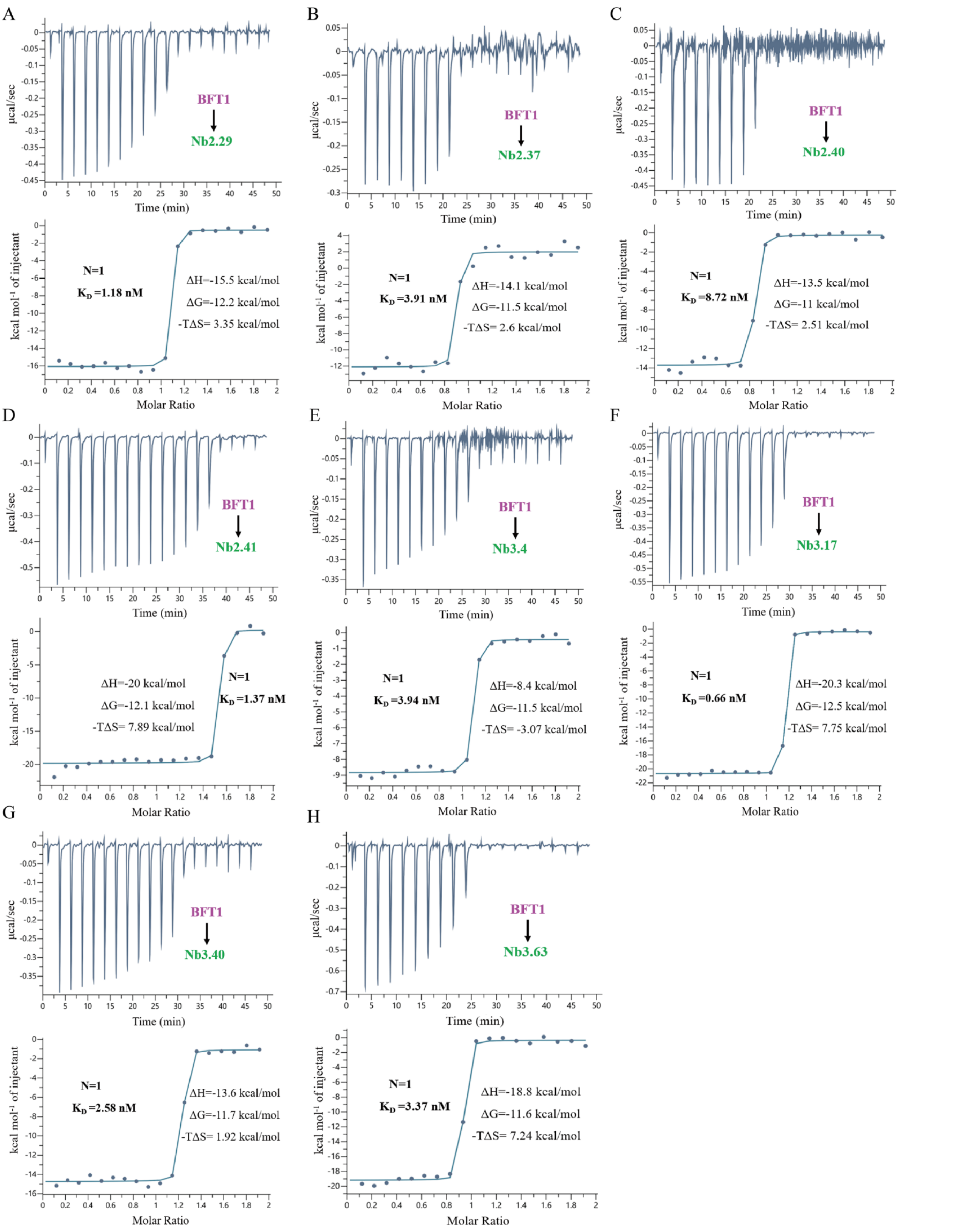
A
µcal/sec
Time (min)
BFT1
Nb2.29
kcal mol-1 of injectant
Molar Ratio
N=1
KD =1.18 nM
ΔH=-15.5 kcal/mol
ΔG=-12.2 kcal/mol
-TΔS= 3.35 kcal/mol
B
BFT1
Nb2.37
N=1
KD =3.91 nM
ΔH=-14.1 kcal/mol
ΔG=-11.5 kcal/mol
-TΔS= 2.6 kcal/mol
C
BFT1
Nb2.40
N=1
KD =8.72 nM
ΔH=-13.5 kcal/mol
ΔG=-11 kcal/mol
-TΔS= 2.51 kcal/mol
D
BFT1
Nb2.41
ΔH=-20 kcal/mol
ΔG=-12.1 kcal/mol
-TΔS= 7.89 kcal/mol
N=1
KD =1.37 nM
E
BFT1
Nb3.4
N=1
KD =3.94 nM
ΔH=-8.4 kcal/mol
ΔG=-11.5 kcal/mol
-TΔS= -3.07 kcal/mol
F
BFT1
Nb3.17
N=1
KD =0.66 nM
ΔH=-20.3 kcal/mol
ΔG=-12.5 kcal/mol
-TΔS= 7.75 kcal/mol
G
BFT1
Nb3.40
N=1
KD =2.58 nM
ΔH=-13.6 kcal/mol
ΔG=-11.7 kcal/mol
-TΔS= 1.92 kcal/mol
H
BFT1
Nb3.63
N=1
KD =3.37 nM
ΔH=-18.8 kcal/mol
ΔG=-11.6 kcal/mol
-TΔS= 7.24 kcal/mol

Supplementary Figure 3. The interactions of nanobodies with BFT1 by isothermal titration calorimetry. **(A)** Nb2.29 **(B)** Nb2.37 **(C)** Nb2.40 **(D)** Nb2.41 **(E)** Nb3.4 **(F)** Nb3.17 **(G)** Nb3.40 **(H)** Nb3.63 also have high affinity to BFT1.

Supplementary Table 1. Biochemical properties for 17 evaluated BFT1 nanobodies. BFT1 nanobodies were successfully cloned in pMES4 vector. The Nb yield varied from 2.5 to 34 mg per liter culture after purification by size exclusion chromatography. The theoretical pI and molecular weight of the nanobodies were calculated from its amino acid sequence.

NO	Name	Theoretical pI	MW(Da)	Total amount (mg/liter)
1	Nb2.1	4.84	14097.46	14.0
2	Nb2.13	5.79	13812.26	8.0
3	Nb2.14	6.77	13324.60	34.0
4	Nb2.29	4.93	13239.64	10.0
5	Nb2.37	6.76	13571.99	2.5
6	Nb2.40	8.98	13199.66	21.4
7	Nb2.41	8.64	13222.61	20.5
8	Nb2.55	7.95	13272.63	17.0
9	Nb2.82	9.23	13413.95	12.7
10	Nb3.4	9.23	12823.32	7.7
11	Nb3.17	6.92	13349.82	7.0
12	Nb3.27	7.94	13270.71	12.6
13	Nb3.40	6.41	13350.63	24.0
14	Nb3.43	8.98	13191.60	24.0
15	Nb3.63	9.01	13948.40	16.8
16	Nb3.74	8.98	13296.72	13.0
17	Nb3.93	8.43	13747.28	8.0

Supplementary Table 2. Summary of interaction details between BFT1 and Nb2.82.

Number	Atom1 in Nb	Distance (Å)	Atom2 in BFT1
		**Hydrogen bonds**	
1	A:ARG 31[NH1]	2.94	C:ASP 169[OD1]
2	A:ARG 31[NH2]	3.55	C:ILE 170[O]
3	A:TYR 32[OH]	2.42	C:ASP 169[OD1]
4	A:THR 33[N]	3.45	C:GLU 128[OE1]
5	A:THR 33[N]	2.98	C:GLN 130[OE1]
6	A:THR 33[OG1]	2.75	C:GLU 128[OE1]
7	A:ASN 52[ND2]	3.86	C:GLU 128[OE2]
8	A:SER 53[N]	2.87	C:GLU 128[OE1]
9	A:SER 53[OG]	2.65	C:GLU 128[OE2]
10	A:ARG 57[NE]	3.40	C:GLU 108[OE2]
11	A:ARG 57[NH2]	3.47	C:ASN 87[O]
12	A:ARG 101[NH1]	3.82	C:GLN 130[O]
13	A:ARG 101[NH2]	3.05	C:GLU 137[OE1]
14	A:TYR 103[OH]	2.93	C:GLU 134[OE1]
15	A:TYR 103[OH]	2.83	C:GLU 134[OE2]
16	A:ARG 31[O]	2.82	C:ALA 129[N]
17	A:THR 33[O]	2.79	C:GLN 130[NE2]
18	A:ASP 54[OD2]	2.98	C:LYS 127[NZ]
19	A:PRO 99[O]	3.07	C:GLN 130[NE2]
		**Salt bridge**	
1	A:ARG 31[NH1]	2.94	C:ASP 169[OD1]
2	A:ARG 57[NE]	3.40	C:GLU 108[OE2]
3	A:ARG 57[NH2]	3.88	C:GLU 108[OE2]
4	A:ARG 101[NH2]	3.05	C:GLU 137[OE1]
5	A:ARG 101[NH2]	3.90	C:GLU 137[OE2]
6	A:ASP 54[OD2]	2.98	C:LYS 127[NZ]

Supplementary Table 3. Summary of interaction details between BFT1 and Nb3.27.

Number	Atom1 in Nb	Distance (Å)	Atom2 in BFT1
		**Hydrogen bonds**	
1	E:GLN 27[N]	3.35	C:ASP 360[OD2]
2	E:THR 28[N]	2.80	C:ASP 360[OD2]
3	E:TRP 32[NE1]	2.73	C:ASP 360[OD1]
4	E:TRP 53[N]	2.88	C:GLU 379[OE1]
5	E:TRP 53[NE1]	3.15	C:ASP 383[OD2]
6	E:ARG 57[NH2]	2.81	C:GLU 207[OE1]
7	E:GLY 101[N]	2.84	C:LEU 377[O]
8	E:TYR 103[N]	3.11	C:GLU 379[OE2]
9	E:TYR 104[OH]	2.50	C:ASP 397[OD2]
10	E:LYS 109[NZ]	3.27	C:ASN 361[OD1]
11	E:THR 28[OG1]	2.96	C:ASP 360[N]
12	E:ALA 31[O]	2.76	C:SER 378[OG]
13	E:ALA 31[O]	3.30	C:GLU 379[N]
14	E:ALA 31[O]	3.25	C:LYS 380[N]
15	E:SER 30[OG]	3.49	C:LYS 380[NZ]
		**Salt bridge**	
1	E:ARG 57[NH2]	2.81	C:GLU 207[OE1]
